# Supplementary material for: A Modified Meiotic Recombination in Brassica napus Largely Improves Its Breeding Efficiency
Source: Biology (Basel). 2021 Aug 13;10(8):771. doi: 10.3390/biology10080771 (PMC8389541; doi:10.3390/biology10080771)
Supplement: Supplementary file 1 [file biology-10-00771-s001.zip › biology-1317319-supplementary.pdf]

Table S1. Meiotic behavior of ArArCoCo-S0 synthetic and AnArCnCo, AnArCn F1 hybrids

Origin	Plant	2n	Number of Pollen Mother cells	Average meiotic behavior				% cells with 19 bivalents
				Univalents	Bivalents	Trivalents	Quadrivalents	
ArArCoCo-S0	ChEM	38	20	0.6	18.65		0.05	70
AnAnCnCn x ArArCoCo	18DChEM 1	38	20	0.2	18.3		0.3	65
	18DChEM 2	38	23	0.18	18.65		0.13	78.26
	18DChEM 3	38	20	0.2	17.9		0.5	50
	18DChEM 4	38	20	0.4	18.7		0.05	75
	18DChEM 5	38	20	0.3	18.45		0.2	65
	18DChEM 7	38	21	0.38	18.43		0.19	61.91
	**18DChEM 8***	38	20	0.2	18.8		0.05	85
	18DChEM 9	38	21	0.57	18.52		0.1	66.67
	18DChEM 10	38	20	0.35	18.65		0.1	75
								% cells with 9 univalents + 10 bivalents
AnAnCnCn x ArAr	18DCh 1	29	20	8.6	10.2			80
	18DCh 2	29	20	8.1	10.45			65
	18DCh 3	29	20	8.7	10.15			75
	18Dch 4	29	20	8.85	10.00	0.05		60
	**18DCh 5***	29	22	9.18	9.18			81.82
	18DCh 6	29	20	8.5	10.25			55
	**18DCh 7***	29	21	9.38	9.81			80.95
	18DCh 8	29	20	8.7	10.15			80.5
	18DCh 9	29	22	8.91	10.05			68.18
	18DCh 10	29	20	8.85	10.00	0.05		80

* plants retained to produce the backcross progeny

Table S2. Correlation metrics between the relative size of introgressions (%) and their relative position to the centromere (%). The *P*-value for the regression analyses are indicated by: ND (Not Determined); p< 0.05: *; p< 0.01: **; p< 0.001: ***.

Chromosomes	Parameters	AnArCn	AnArCnCo
A01	R^2	0.93 ***	0.98 ***
	Equation	$y=-0.23x+0.33$	$y=-0.35x^2+0.22x+0.4$
A02	R^2	0.83 ***	0.92 ***
	Equation	$y=-0.12x+0.28$	$y=-0.41x^2+0.29x+0.4$
A03	R^2	ND	ND
	Equation	ND	ND
A04	R^2	0.83 ***	0.90 ***
	Equation	$y=-0.12x+0.28$	$y=-0.2x^2+0.11x+0.4$
A05	R^2	0.96 ***	0.97 ***
	Equation	$y=-0.37x+0.41$	$y=-0.56x^2+0.41x+0.37$
A06	R^2	0.92 ***	0.95 ***
	Equation	$y=-0.27x+0.35$	$y=-0.59x^2+0.44x+0.38$
A07	R^2	0.69 ***	0.69 ***
	Equation	$y=-0.11x+0.29$	$y=-0.17x^2+0.1x+0.38$
A08	R^2	0.80 ***	0.79 ***
	Equation	$y=-0.25x+0.38$	$y=-0.39x^2+0.24x+0.4$
A09	R^2	0.90 ***	0.92 ***
	Equation	$y=-0.2x+0.28$	$y=-0.37x^2+0.26x+0.33$
A10	R^2	0.92 ***	0.55 ***
	Equation	$y=-0.17x+0.36$	$y=-0.12x^2-0.01x+0.45$
A genome	R^2	0.66 ***	0.68 ***
	Equation	$y=-0.2x+0.33$	$y=-0.33x^2+0.21x+0.39$

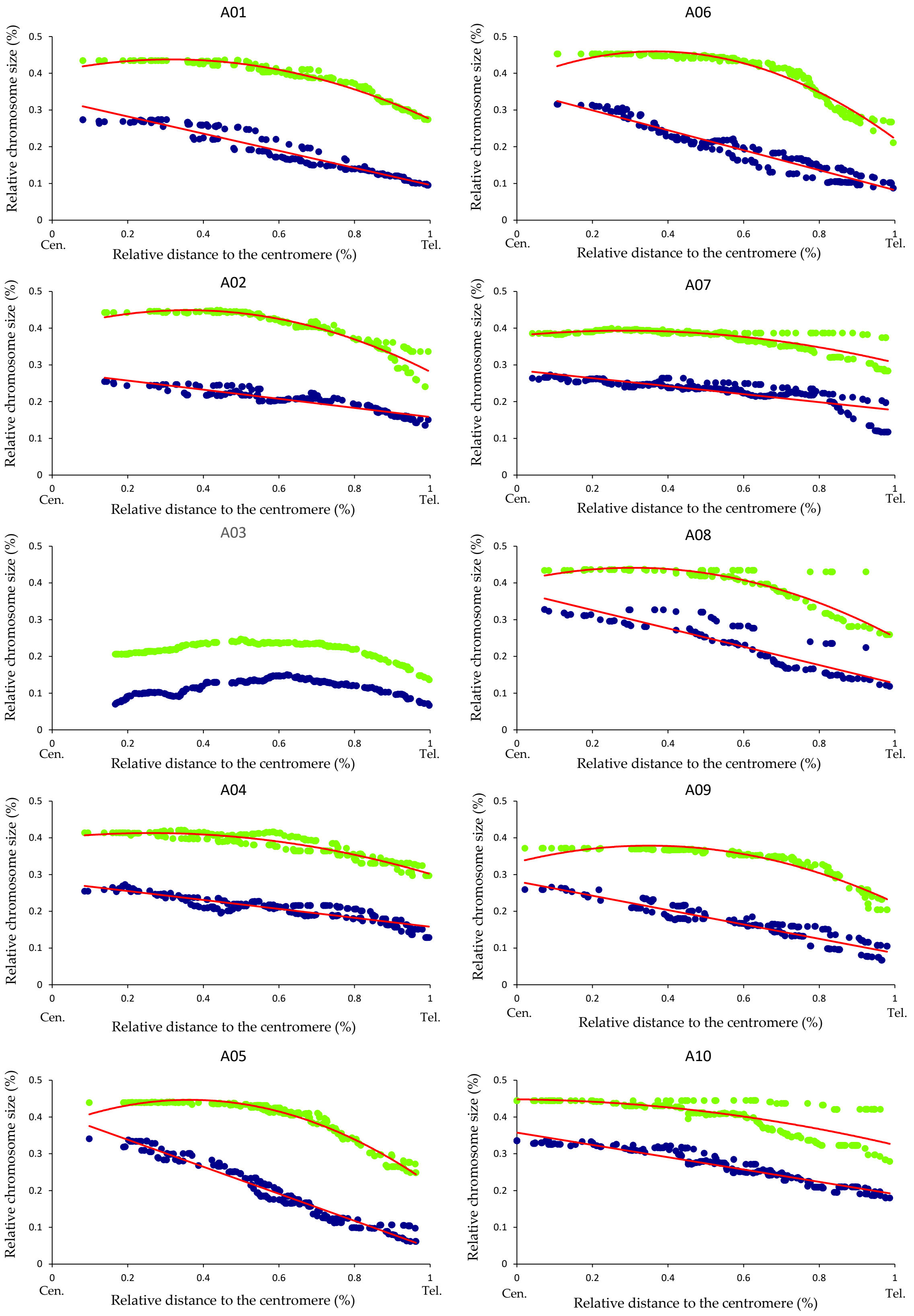

Figure S1. Correlations between the relative size of introgressions and their relative position from the centromere (Cen.) to the telomere (Tel.), analyzed in the progeny of the AnArCn hybrid (blue) and AnArCnCo hybrid (green) for the ten A chromosomes.Regressions lines are indicated in red.
